# Sex Differences in the Impact of Body Mass Index on the Risk of Future Atrial Fibrillation: Insights From the Longitudinal Population‐Based Tromsø Study

**DOI:** 10.1161/JAHA.117.008414

**Published:** 2018-04-19

**Authors:** Jocasta Ball, Maja‐Lisa Løchen, Tom Wilsgaard, Henrik Schirmer, Laila A. Hopstock, Bente Morseth, Ellisiv B. Mathiesen, Inger Njølstad, Sweta Tiwari, Ekaterina Sharashova

**Affiliations:** ^1^ Pre‐Clinical Disease and Prevention Baker Heart and Diabetes Institute Melbourne Australia; ^2^ School of Public Health and Preventive Medicine Monash University Melbourne Australia; ^3^ Department of Community Medicine UiT The Arctic University of Norway Tromsø Norway; ^4^ Department of Clinical Medicine UiT The Arctic University of Norway Tromsø Norway; ^5^ Department of Health and Care Sciences UiT The Arctic University of Norway Tromsø Norway; ^6^ School of Sport Sciences UiT The Arctic University of Norway Tromsø Norway; ^7^ Department of Clinical Medicine University of Oslo Norway; ^8^ Department of Cardiology Akershus University Hospital Lørenskog Norway; ^9^ Department of Neurology University Hospital of North Norway Tromsø Norway

**Keywords:** atrial fibrillation, body mass index, incidence, sex differences, Epidemiology, Atrial Fibrillation, Risk Factors

## Abstract

**Background:**

Atrial fibrillation (AF) prevalence is increasing, and body mass index (BMI) is a risk factor for AF. However, sex differences in the impact of BMI on AF risk have not been fully elucidated.

**Methods and Results:**

Data from the fourth survey (1994–1995) of the Tromsø Study (Norway) were used to investigate the association of single‐measurement BMI on future AF risk. To analyze the influence of BMI changes on AF risk, data from individuals who attended the third and fourth study surveys were used. AF diagnosis was derived from record linkage and end point adjudication. Cox regression analysis was conducted using fractional polynomials of BMI and BMI change with models adjusted for age, baseline BMI (change analyses), risk factors, comorbidities, and antihypertensive medications.

Data were available for 24 799 individuals from the fourth survey (mean age, 45.5±14.2 years; 52.9% women). Over 15.7±5.5 years, 811 women (6.2%) and 918 men (7.9%) developed AF. In men, lower BMI decreased AF risk and higher BMI increased risk (hazard ratios [95% confidence intervals] for BMI 18 or 40 kg/m^2^ compared with 23 kg/m^2^ were 0.75 [0.70–0.81] and 4.42 [3.00–6.53], respectively). The same pattern was identified in women. Two surveys were attended by 14 652 individuals. In men and women, a decrease in BMI over time was associated with decreased AF risk and an increase in BMI was associated with increased AF risk.

**Conclusions:**

Within a population cohort, BMI was positively associated with AF risk. Change in BMI over time influenced AF risk in both men and women.

**Clinical Perspective**

**What Is New?**

- Data from the Tromsø Study showed that in men and women, lower body mass index (BMI) was associated with a decreased risk of future atrial fibrillation (AF) development and increased BMI was associated with increased AF risk.
- In men and women, a decrease in BMI over time was associated with a decreased risk of future AF and an increase in BMI over the same time was associated with increased AF risk.
- Sex differences were identified in the risk of AF development, with associations found to be stronger in men than in women for both single‐measurement BMI and BMI change over time.

**What Are the Clinical Implications?**

- Higher BMI potentially has a greater influence on future AF development in men than in women.
- Identifying sex‐specific differences in the influence of BMI on incident AF will assist in the primary prevention of AF and has the potential to contribute to sex‐specific preventive strategies.
- Interventions to decrease BMI should be encouraged for men and women with higher BMI above that considered normal.
- Even modest reductions in population BMI are likely to have a significant effect on the public health burden of AF.

Atrial fibrillation (AF) is the most common cardiac rhythm disturbance observed and treated in clinical practice. In 2010, an estimated 5 million incident cases of AF were diagnosed globally, while AF is prevalent in 2% to 3% of the world's population overall.^1^, ^2^ Prevalence of AF is increasing in parallel to population ageing and improved survival from other clinical conditions that increase the likelihood of AF development. Furthermore, little progress has been made in reducing major significant risk factors for AF, such as excess body weight.^2^ Multiple complications are the direct result of AF, including thromboembolic events (notably stroke), heart failure, reduced quality of life, and even death.^3^, ^4^

Over recent decades, the global prevalence of overweight and obesity has increased rapidly to epidemic proportions.^5^ In northern Norway, population trends in mean body mass index (BMI) and the prevalence of obesity have demonstrated a steady increase over time, particularly in younger age groups.^6^ Overweight and obesity are significant risk factors for multiple chronic diseases, including cardiovascular disease (CVD), diabetes mellitus, cancer, and musculoskeletal disorders. Excess body weight causes ≈3 million deaths around the world every year.^7^ Current epidemiological trends in overweight and obesity are major public health challenges, particularly in relation to reduced quality of life and increased health costs.^8^

It is already established that overweight and obesity are associated with an increased risk of AF, although lack of consensus about patterns of association exists.^9^, ^10^ Some have suggested a J‐shaped dose‐response relationship between BMI and AF^11^, ^12^; however, others have found a linear association.^13^, ^14^, ^15^, ^16^, ^17^, ^18^, ^19^ A recent systematic review and dose‐response meta‐analysis of prospective studies demonstrated a nonlinear association between BMI and risk of AF, with a stronger association at higher BMI levels and a 28% increase in the relative risk of AF for every 5 kg/m^2^ increase in BMI identified.^20^ Increased risk was also demonstrated with relatively higher BMI, even within the normal BMI range.^20^ However, not all included studies took sex into consideration during analysis. The incidence of AF differs between men and women (it is up to 2 times higher in men^21^) because of the prevalence of major risk factors for AF, including hypertension, diabetes mellitus, coronary artery disease, and heart failure, also differing between the sexes.^21^ Given that excess body weight is a modifiable risk factor and AF differs causatively in women versus men,^22^ it is important to understand if differences in the influence of BMI exist between men and women. We, therefore, aimed to investigate the impact of BMI on longer‐term risk of AF and the impact of change in BMI over time in men and women using the longitudinal Tromsø Study.

## Methods

The data, analytic methods, and study materials will not be made available to other researchers for purposes of reproducing the results or replicating the procedure.

### Study Setting and Participants

**Figure 1:**
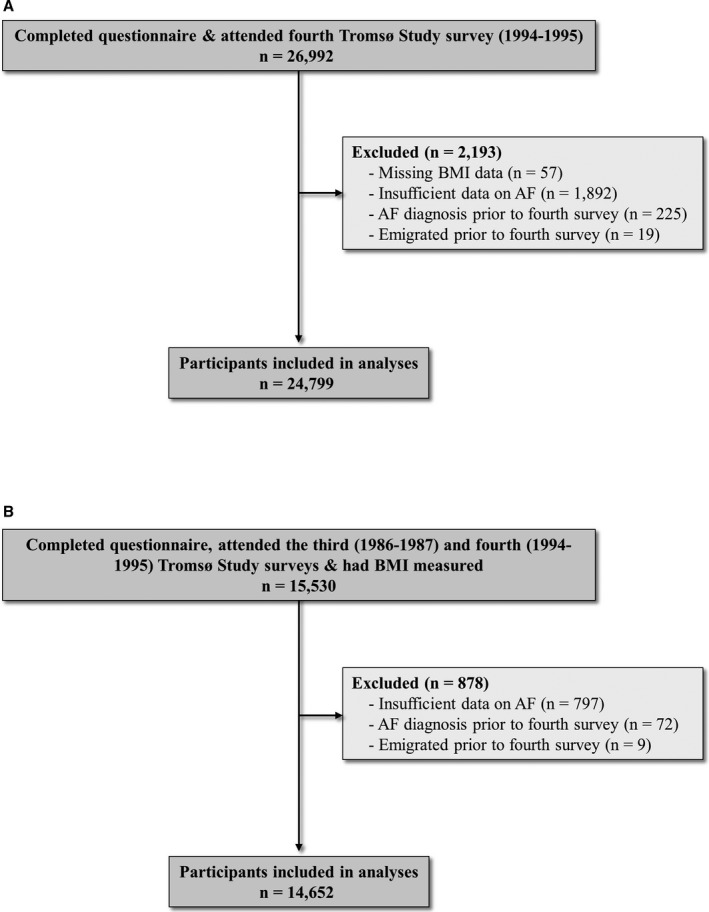
Study flow chart of included participants for analyses of single‐measurement body mass index (BMI) (A) and individual change in BMI over time (B), in the Tromsø Study (1986–1995). AF indicates atrial fibrillation.

The Tromsø Study is a population‐based longitudinal study involving CVD epidemiological screening, the details of which have been described previously.^23^, ^24^ In brief, total birth cohorts in addition to random population samples from the municipality of Tromsø, northern Norway, were invited to participate in ≥1 of 7 health surveys conducted from 1974 to 2016. For the current study, data were used from participants who attended the third (1986–1987) and fourth (1994–1995) surveys. Each study visit was conducted by trained personnel and according to the same core protocol. Data collection consisted of questionnaires, interviews, physical measurements, biological sample collection, and clinical examinations.^23^, ^24^ Data from individuals who had missing information on BMI, who emigrated before the start of follow‐up, or who developed AF before the start of follow‐up were excluded from the current analyses. Individuals for whom outcome data were insufficient to confirm or deny a diagnosis of AF were also excluded from analyses. Figure 1A demonstrates the total cohort who attended the fourth Tromsø Study survey, consisting of 24 799 individuals (11 673 men and 13 126 women; mean age, 45.5±14.2 years) included in the analysis of BMI influence on risk of future AF. The same data for the cohort included in analyses of individual change in BMI over time and influence on future AF (n=14 652) are also included in Figure 1B. For this change analysis, individuals who attended the third and fourth Tromsø Study surveys were included (7158 men and 7494 women).

#### Ethical considerations

The Tromsø Study was approved by the Data Inspectorate and by the Regional Committee for Medical Research Ethics, North Norway, and was performed in accordance with the ethical standards laid down in the Declaration of Helsinki. All participants from the fourth survey provided informed consent, and living participants from prior surveys were provided the opportunity to withdraw from the study.

### Data Collection

#### Cohort profiling

Use of antihypertensive medication (yes/no), leisure time physical activity (inactive/low activity/moderate activity/high activity), current daily smoking (yes/no), and medical history (myocardial infarction, angina pectoris, stroke, diabetes mellitus; yes/no) were extracted from the Tromsø Study questionnaire. Physical activity levels were determined by the number of self‐reported hours per week of light activity and hard activity. Questions on leisure time physical activity were different in the fourth survey from those in the prior survey; therefore, answers were recoded to correspond to the categories used in the third survey.^25^

Nonfasting serum total cholesterol, high‐density lipoprotein (HDL) cholesterol, and triglyceride levels (mmol/L) were analyzed by the Department of Laboratory Medicine, University Hospital of North Norway (Tromsø). Blood pressure was measured using an oscillometric digital automatic device (Dinamap Vital Signs Monitor 1846; Critikon Inc, Tampa, FL) with the mean of the last 2 of 3 recordings used. Weight and height were measured with only light clothing on and no shoes and were used to calculate BMI (kilograms per meter squared [kg/m^2^]). The World Health Organization international classification of BMI groups (including additional cutoffs for public health action) was used to categorize the participant cohort.^26^ Participants were classified as being underweight (BMI, <18.50 kg/m^2^), normal weight (BMI, 18.50–22.99 kg/m^2^; or BMI, 23.00–24.99 kg/m^2^), overweight (BMI, 25.00–27.49 kg/m^2^; or BMI, 27.50–29.99 kg/m^2^), or obese (class I: BMI, 30.00–34.99 kg/m^2^; or class II/III: BMI, ≥35.00 kg/m^2^).^26^

#### Follow‐up and incident AF detection

Follow‐up began from the date of the last Tromsø Study survey examination attended (1994–1995) until the date of first documented AF, date of censorship because of migration or death (identified through the Population Register of Norway), or the end of follow‐up at December 31, 2013, whichever came first. Incident cases of AF documented on ECG were derived from hospital record (diagnosis registry including outpatient clinic) linkage with the University Hospital of North Norway (the only hospital within a radius of 250 km) and to the National Causes of Death Registry using the following diagnostic codes: *International Classification of Diseases, Ninth Revision* (*ICD‐9*) codes 427.0 to 427.99 and *International Classification of Diseases, Tenth Revision* (*ICD‐10*) codes I47 and I48. Norway has a unique personal identification system that makes exact matching in medical registers possible. For participants with diagnoses of cerebrovascular or cardiovascular events, hospital records were also reviewed for further AF events. Adjudication of all AF events was conducted by an end point committee.^27^ AF subtype was categorized into any, paroxysmal/persistent, or permanent.^28^ According to management guidelines, AF is classified as being paroxysmal (lasting ≤7 days and self‐terminating), persistent (lasting >7 days, usually requiring termination with cardioversion and/or medication), or permanent (for which a rhythm control strategy is not pursued).^28^ Within the current study, 49.6% of men and 49.5% of women developed paroxysmal or persistent AF, and 45.9% of men and 43.7% of women developed permanent AF during follow‐up. The pattern of AF was unknown in 4.6% of men and 6.9% of women. AF occurring during the last 7 days of life, postoperative AF occurring within 28 days after surgery, AF related to myocardial infarction or within 28 days after myocardial infarction, and AF related to other acute cardiac events (eg, acute heart failure or pulmonary edema) or within 28 days after an event were classified as noncases.

### Statistical Analyses

A significant interaction between single‐measurement BMI and sex was identified (*P*=0.008); therefore, all analyses were stratified by sex. No interaction was found between BMI and age in women, but an interaction was found in men (*P*=0.025). No interaction between BMI change and baseline BMI, while adjusting for baseline age, was identified in either men or women. Descriptive data are presented as mean±SD for continuous variables or as number and proportion (%) for categorical variables. Between‐group comparisons were conducted with linear regression, logistic regression, linear mixed models, or generalized estimating equations, where appropriate, and all models were adjusted for age. All data were analyzed and forest plots generated using Statistical Analysis Software, version 9.4 (SAS Institute Inc, Cary, NC). *P*<0.05 (2‐sided) was considered statistically significant.

#### Analysis of single‐measurement BMI on risk of future AF

Cox proportional hazard regression analysis was conducted (with calculation of hazard ratios [HRs] and 95% confidence intervals [CIs]) to assess the association of single‐measurement BMI on the risk of future AF, with AF as the dependent variable, fractional polynomials of BMI as the main predictor, and fractional polynomials of age as a covariate. A BMI of 23.0 kg/m^2^ was used as the reference. The model was additionally adjusted for systolic blood pressure; total cholesterol, HDL cholesterol, and triglyceride levels; current daily smoking; physical activity levels; CVD (myocardial infarction, angina, and stroke); concurrent diabetes mellitus; and current antihypertensive medications. Stratification of results by age (<65 versus ≥65 years) was conducted for men because of the demonstrated interaction between BMI and age. Sensitivity analyses were conducted with the systematic removal of potential mediating factors (comorbidities, biomedical risk factors, and health‐related behaviors) for the association between BMI and incident AF. Sensitivity analyses were also conducted to investigate the impact of AF type (paroxysmal/persistent versus permanent) on the association of single‐measurement BMI on the future risk of AF.

#### Analysis of individual 10‐year change in BMI on risk of future AF

Individual change in BMI was estimated as the slope of the linear regression model, with BMI measured in the third and fourth Tromsø Study surveys as the dependent variable and year of examination as the predictor. Cox proportional hazard regression analysis was conducted (with calculation of HRs and 95% CIs) to assess the impact of individual change in BMI over 10 years (slopes multiplied by 10) on the risk of future AF, with AF as the dependent variable, fractional polynomials of BMI change over 10 years as the main predictor, and fractional polynomials of baseline BMI and fractional polynomials of baseline age as covariates. An increase in BMI of 1 unit (1 kg/m^2^) per 10 years was used as the reference. The model was additionally adjusted for baseline values of systolic blood pressure; total cholesterol, HDL cholesterol, and triglyceride levels; current daily smoking; physical activity levels; CVD (myocardial infarction, angina, and stroke); concurrent diabetes mellitus; and current antihypertensive medications. As with data on the single measurement of BMI, sensitivity analyses were conducted to investigate the impact of AF type on the association of BMI change over 10 years on the future risk of AF.

## Results

### Baseline Characteristics

Table 1 demonstrates the baseline characteristics of the total included cohort of 24 799 men and women who attended the fourth Tromsø Study survey, according to BMI classification groups.^26^ Among men, the BMI groups differed with respect to age, risk factors for CVD, history of CVD, and self‐reported antihypertensive medication use. Mean age differed across the BMI groups, with underweight men being the oldest (mean age, 50.9±17.7 years) and those with a BMI ≥35.00 kg/m^2^ being slightly younger (mean age, 46.4±13.5 years) than those with a BMI between 27.50 and 34.99 kg/m^2^. Mean systolic blood pressure and total cholesterol and triglyceride levels all increased linearly with increasing BMI. HDL levels decreased linearly as BMI increased. Men with a BMI up to 27.49 kg/m^2^ were more likely to report undertaking “moderate” leisure time physical activity than those with a BMI ≥27.50 kg/m^2^, who were more likely to report undertaking “low” physical activity. Underweight men were more likely to report being a smoker (71.8%), with linearly decreasing prevalence as BMI increased. The proportion of men reporting a previous myocardial infarction increased with increasing BMI. However, the highest proportion of individuals who had experienced a previous stroke were in the underweight group (2.2%). As expected, the proportion of men with diabetes mellitus increased with increasing BMI, as did the proportion of men who were prescribed antihypertensive medications.

**Table 1:** Baseline Characteristics of 11 673 Men and 13 126 Women According to BMI Groups: The Tromsø Study, 1994 to 1995

Characteristics	BMI, kg/m^2^							*P* Value
	Underweight	Normal Weight		Overweight		Obese		
	<18.50	18.50–22.99	23.00–24.99	25.00–27.49	27.50–29.99	30.00–34.99	≥35.00	
Men								
No.	62	2385	2973	3406	1775	940	132	
Age, mean (SD), y	50.9 (17.7)	42.5 (13.8)	43.6 (13.2)	46.2 (13.5)	47.7 (13.3)	47.6 (13.2)	46.4 (13.5)	<0.001
Systolic blood pressure, mean (SD), mm Hg	128.5 (16.7)	129.7 (15.0)	131.9 (15.1)	134.3 (16.1)	138.0 (17.4)	139.1 (17.6)	142.4 (19.5)	<0.001
Total cholesterol, mean (SD), mmol/L	5.43 (1.17)	5.67 (1.18)	5.91 (1.18)	6.13 (1.19)	6.29 (1.20)	6.28 (1.13)	6.40 (1.16)	<0.001
HDL cholesterol, mean (SD), mmol/L	1.59 (0.36)	1.46 (0.37)	1.39 (0.34)	1.32 (0.34)	1.24 (0.31)	1.18 (0.30)	1.08 (0.23)	<0.001
Triglycerides, mean (SD), mmol/L	1.12 (0.54)	1.31 (0.76)	1.53 (0.89)	1.86 (1.23)	2.20 (1.30)	2.53 (1.58)	2.90 (1.56)	<0.001
Physical activity, n (%)								
Inactive	11 (15.3)	195 (8.4)	195 (6.6)	224 (6.2)	168 (8.8)	105 (10.4)	17 (12.5)	<0.001
Low activity	23 (37.7)	860 (36.1)	1086 (36.7)	1367 (40.6)	768 (44.1)	410 (44.4)	59 (45.9)	<0.001
Moderate activity	26 (41.3)	1059 (45.0)	1338 (45.6)	1469 (43.3)	692 (39.2)	362 (38.6)	48 (37.1)	<0.001
High activity	2 (3.4)	252 (9.8)	325 (10.4)	319 (9.4)	124 (7.2)	53 (5.8)	5 (3.8)	<0.001
Current smoking, n (%)	44 (71.8)	1181 (49.2)	1106 (37.0)	1157 (34.1)	570 (32.5)	295 (31.7)	35 (26.7)	<0.001
History of myocardial infarction, n (%)	0 (0.0)	39 (0.8)	63 (1.1)	111 (1.4)	80 (1.8)	53 (2.4)	7 (2.5)	<0.001
History of angina pectoris, n (%)	1 (0.3)	45 (0.8)	83 (1.3)	126 (1.4)	102 (2.1)	65 (2.7)	11 (3.6)	<0.001
History of stroke, n (%)	4 (2.2)	22 (0.6)	27 (0.6)	38 (0.6)	28 (0.8)	21 (1.1)	1 (0.4)	0.040
Diabetes mellitus, n (%)	0 (0.0)	17 (0.5)	27 (0.6)	31 (0.5)	32 (1.0)	30 (1.7)	8 (3.7)	<0.001
Antihypertensive medications, n (%)	3 (1.5)	53 (1.4)	90 (1.9)	176 (2.8)	135 (4.0)	106 (6.4)	22 (11.0)	<0.001
Women								
No.	269	4901	2812	2415	1314	1126	289	
Age, mean (SD), y	42.3 (15.5)	41.4 (12.8)	45.3 (14.0)	48.6 (14.8)	51.5 (15.8)	53.0 (15.3)	52.5 (15.3)	<0.001
Systolic blood pressure, mean (SD), mm Hg	122.6 (17.1)	123.9 (16.5)	126.2 (19.2)	128.9 (21.0)	133.4 (23.2)	137.3 (23.4)	142.7 (24.2)	<0.001
Total cholesterol, mean (SD), mmol/L	5.70 (1.21)	5.75 (1.24)	6.00 (1.32)	6.17 (1.38)	6.25 (1.38)	6.36 (1.32)	6.31 (1.37)	<0.001
HDL cholesterol, mean (SD), mmol/L	1.73 (0.43)	1.73 (0.40)	1.66 (0.40)	1.61 (0.40)	1.54 (0.40)	1.45 (0.36)	1.41 (0.37)	<0.001
Triglycerides, mean (SD), mmol/L	1.06 (0.54)	1.07 (0.54)	1.25 (0.73)	1.42 (0.89)	1.65 (1.03)	1.87 (1.14)	1.92 (1.03)	<0.001
Physical activity, n (%)								
Inactive	31 (11.0)	333 (6.9)	212 (6.4)	211 (6.5)	150 (7.6)	173 (10.1)	58 (13.9)	<0.001
Low activity	105 (39.5)	1983 (40.6)	1167 (42.0)	1045 (44.0)	623 (48.3)	523 (47.7)	139 (49.2)	<0.001
Moderate activity	121 (45.0)	2272 (45.9)	1286 (46.3)	1047 (44.7)	497 (39.7)	392 (37.1)	80 (29.5)	<0.001
High activity	8 (2.6)	266 (4.8)	118 (4.0)	85 (3.7)	30 (2.5)	21 (2.2)	8 (3.2)	<0.001
Current smoking, n (%)	154 (56.5)	2128 (42.0)	1026 (36.5)	787 (33.6)	383 (31.0)	302 (29.1)	70 (26.1)	<0.001
History of myocardial infarction, n (%)	4 (0.6)	21 (0.2)	20 (0.3)	31 (0.4)	28 (0.5)	25 (0.5)	5 (0.4)	0.132
History of angina pectoris, n (%)	5 (0.5)	53 (0.4)	55 (0.5)	82 (0.7)	75 (0.9)	63 (0.9)	22 (1.3)	<0.001
History of stroke, n (%)	2 (0.4)	34 (0.5)	23 (0.5)	20 (0.4)	15 (0.4)	22 (0.7)	7 (0.9)	0.361
Diabetes mellitus, n (%)	1 (0.3)	25 (0.5)	20 (0.5)	36 (0.8)	27 (1.0)	43 (1.7)	21 (3.6)	<0.001
Antihypertensive medications, n (%)	6 (1.3)	83 (1.3)	94 (1.9)	159 (3.1)	138 (4.1)	155 (5.3)	50 (7.4)	<0.001

Values are mean (SD) or number (percentage); the means (except age means) and percentages are adjusted for age using linear or logistic regression models, respectively. Because of missing observations, numbers for the variables may be marginally less. BMI indicates body mass index; HDL, high‐density lipoprotein.

In women, the patterns identified were the same as for men. Similarly to the men, the age of women differed across the BMI groups, with the highest mean age demonstrated in obese class I (BMI, 30.00–34.99 kg/m^2^) individuals. Patterns in blood pressure and cholesterol levels mirrored those identified in the male cohort, as did patterns of physical activity and smoking rates, with women in the underweight group reporting the highest proportion of smokers (56.5%). History of myocardial infarction was not significantly different between individuals in the different BMI groups. In contrast to the male cohort, women with a BMI ≥35.00 kg/m^2^ reported the highest rate of stroke (0.9%), although differences were not significantly different. The proportion of participants with diabetes mellitus and those reporting antihypertensive medication use increased linearly with increasing BMI. Tables 2 and 3 demonstrate the baseline profile of men (n=7158) and women (n=7494) included in the analyses of the impact of 10‐year change in BMI on the risk of future AF. Some patterns in risk factors and incident CVD were similar in men and women, with significant differences demonstrated between the different BMI change categories for most variables. However, men demonstrated higher systolic blood pressure, lower HDL levels, and higher triglyceride levels than women. Furthermore, men were more active than women and had more incident cases of myocardial infarction and angina pectoris between the 2 surveys than women.

**Table 2:** Baseline Characteristics of 7158 Men According to BMI Change Groups: The Tromsø Study

Characteristics	10‐y Change in BMI, kg/m^2^						*P* Value
	<−1	−1 to <0	0 to <1	1 to <2	2 to <3	≥3	
Participants, n	666	976	1548	1600	1118	1250	
AF cases, n (%)^a^	62 (3.8)	80 (3.7)	128 (4.6)	128 (4.6)	85 (5.2)	92 (5.9)	0.036
Age, mean (SD), y							
1986–1987	42.4 (11.3)	41.8 (10.7)	39.6 (10.3)	39.4 (10.2)	37.6 (10.0)	35.6 (10.3)	<0.001
At AF diagnosis	39.5 (5.3)	41.1 (4.7)	40.7 (4.8)	40.8 (4.8)	40.4 (4.7)	39.9 (5.2)	0.298
BMI, mean (SD), kg/m^2^							
1986–1987	25.9 (3.7)	24.8 (2.9)	24.5 (2.7)	24.6 (2.7)	24.7 (2.7)	25.1 (3.1)	<0.001
1994–1995	23.7 (3.4)	23.9 (2.9)	24.5 (2.7)	25.4 (2.8)	26.3 (2.7)	28.0 (3.3)	<0.001
Systolic blood pressure, mean (SD), mm Hg							
1986–1987	134.8 (15.8)	133.4 (13.8)	132.6 (13.6)	132.8 (13.5)	132.8 (13.0)	133.9 (13.1)	0.003
1994–1995	130.0 (17.4)	131.1 (17.0)	130.9 (16.3)	132.5 (16.1)	133.2 (15.9)	134.9 (15.7)	<0.001
Total cholesterol, mean (SD), mmol/L							
1986–1987	6.10 (1.23)	6.08 (1.28)	5.97 (1.21)	5.96 (1.20)	5.96 (1.19)	5.96 (1.26)	0.005
1994–1995	5.65 (1.21)	5.79 (1.21)	5.84 (1.15)	5.96 (1.19)	6.07 (1.19)	6.21 (1.17)	<0.001
HDL cholesterol, mean (SD), mmol/L							
1986–1987	1.35 (0.33)	1.38 (0.35)	1.38 (0.32)	1.37 (0.32)	1.37 (0.35)	1.37 (0.33)	0.458
1994–1995	1.43 (0.39)	1.42 (0.39)	1.38 (0.35)	1.33 (0.34)	1.31 (0.33)	1.26 (0.31)	<0.001
Triglycerides, mean (SD), mmol/L							
1986–1987	1.77 (1.13)	1.65 (0.91)	1.59 (0.94)	1.61 (0.96)	1.62 (1.04)	1.62 (1.01)	0.003
1994–1995	1.36 (0.88)	1.50 (0.95)	1.56 (0.97)	1.78 (1.13)	1.96 (1.27)	2.24 (1.41)	<0.001
Physical activity, n (%)							
1986–1987							
Inactive	177 (26.6)	202 (20.7)	295 (19.1)	338 (21.2)	222 (19.9)	292 (23.4)	<0.001
Low activity	352 (52.7)	505 (51.8)	795 (52.1)	803 (51.0)	566 (52.0)	590 (49.2)	0.915
Moderate activity	116 (17.4)	240 (24.6)	387 (24.9)	397 (24.7)	280 (24.8)	306 (24.1)	0.009
High activity	21 (2.9)	29 (2.7)	70 (3.8)	61 (3.2)	50 (3.5)	62 (3.5)	0.710
1994–1995							
Inactive	55 (8.3)	85 (8.8)	102 (6.6)	88 (5.5)	90 (8.1)	106 (8.6)	0.004
Low activity	261 (37.1)	338 (32.8)	593 (37.1)	675 (40.9)	477 (41.9)	586 (46.9)	<0.001
Moderate activity	284 (43.8)	455 (47.8)	677 (44.7)	684 (43.5)	460 (41.8)	466 (37.8)	0.003
High activity	59 (11.4)	87 (11.3)	159 (12.0)	141 (10.2)	82 (8.0)	78 (6.2)	<0.001
Current smoking, n (%)							
1986–1987	310 (46.6)	441 (45.1)	674 (43.0)	662 (40.8)	493 (43.2)	607 (47.3)	0.007
1994–1995	345 (53.6)	425 (45.3)	633 (42.1)	582 (37.5)	370 (33.8)	349 (28.3)	<0.001
History of myocardial infarction, n (%)^a^							
New cases between surveys	18 (1.1)	17 (0.8)	28 (0.9)	28 (1.0)	16 (0.9)	27 (1.7)	<0.001
History of angina pectoris, n (%)^a^							
New cases between surveys	29 (1.4)	31 (1.1)	33 (1.1)	28 (0.8)	16 (0.9)	37 (1.9)	<0.001
History of stroke, n (%)^a^							
New cases between surveys	16 (1.3)	7 (0.5)	15 (0.7)	11 (0.5)	6 (0.5)	6 (0.5)	0.066
Diabetes mellitus, n (%)^a^							
New cases between surveys	17 (1.2)	8 (0.4)	12 (0.5)	4 (0.1)	3 (0.2)	8 (0.5)	<0.001
Antihypertensive medications, n (%)							
1986–1987	36 (3.0)	17 (1.0)	38 (1.8)	47 (2.2)	18 (1.5)	21 (1.8)	0.010
1994–1995	58 (2.2)	45 (1.2)	68 (1.4)	97 (2.1)	44 (1.6)	68 (2.7)	<0.001

Values are mean (SD) or number (percentage); the means (except age means) and percentages are adjusted for age between the surveys and 10‐year BMI change groups using linear mixed models or generalized estimating equations, respectively. AF indicates atrial fibrillation; BMI, body mass index; HDL, high‐density lipoprotein.

a Percentages are adjusted for age in 1986 to 1987 using a logistic regression model. Because of missing observations, numbers for the variables may be marginally less.

**Table 3:** Baseline Characteristics of 7494 Women According to BMI Change Groups: The Tromsø Study

Characteristics	10‐y Change in BMI, kg/m^2^						*P* Value
	<−1	−1 to <0	0 to <1	1 to <2	2 to <3	≥3	
Participants, n	708	773	1255	1450	1202	2106	
AF cases, n (%)^a^	24 (1.8)	21 (1.4)	37 (1.5)	40 (1.5)	43 (2.1)	95 (2.8)	0.003
Age, mean (SD), y							
1986–1987	36.4 (10.4)	37.5 (9.9)	37.6 (9.5)	37.2 (9.4)	37.3 (9.2)	36.7 (9.4)	0.021
At AF diagnosis	40.7 (4.6)	41.0 (3.8)	42.4 (4.6)	41.1 (4.7)	40.2 (5.2)	41.5 (4.7)	0.408
BMI, mean (SD), kg/m^2^							
1986–1987	25.1 (4.0)	23.3 (3.1)	23.0 (3.0)	23.0 (3.0)	23.4 (3.1)	24.2 (3.7)	<0.001
1994–1995	22.3 (3.6)	22.1 (3.1)	22.7 (3.0)	23.4 (3.0)	24.6 (3.1)	27.4 (4.3)	<0.001
Systolic blood pressure, mean (SD), mm Hg							
1986–1987	127.2 (15.6)	124.3 (13.7)	124.2 (13.9)	125.3 (14.2)	125.0 (14.7)	125.8 (14.2)	<0.001
1994–1995	121.8 (18.3)	121.3 (17.1)	121.4 (16.9)	122.4 (17.2)	124.1 (18.1)	127.4 (18.3)	<0.001
Total cholesterol, mean (SD), mmol/L							
1986–1987	6.27 (1.30)	5.99 (1.27)	5.90 (1.28)	5.89 (1.22)	5.85 (1.17)	5.97 (1.23)	<0.001
1994–1995	5.68 (1.29)	5.69 (1.31)	5.66 (1.29)	5.69 (1.25)	5.80 (1.27)	6.06 (1.28)	<0.001
HDL cholesterol, mean (SD), mmol/L							
1986–1987	1.67 (0.39)	1.70 (0.38)	1.69 (0.39)	1.68 (0.38)	1.68 (0.37)	1.65 (0.36)	0.006
1994–1995	1.68 (0.41)	1.70 (0.42)	1.69 (0.41)	1.64 (0.39)	1.63 (0.38)	1.56 (0.37)	<0.001
Triglycerides, mean (SD), mmol/L							
1986–1987	1.44 (0.84)	1.19 (0.69)	1.12 (0.58)	1.14 (0.59)	1.14 (0.59)	1.16 (0.58)	<0.001
1994–1995	1.10 (0.67)	1.10 (0.71)	1.11 (0.70)	1.19 (0.79)	1.25 (0.78)	1.50 (0.92)	<0.001
Physical activity, n (%)							
1986–1987							
Inactive	181 (26.2)	181 (23.9)	277 (22.6)	318 (22.4)	262 (22.3)	520 (25.3)	0.149
Low activity	462 (65.9)	509 (66.4)	841 (67.7)	972 (67.7)	832 (69.8)	1397 (67.0)	0.521
Moderate activity	59 (8.0)	77 (9.7)	118 (9.1)	142 (9.5)	96 (7.7)	175 (8.0)	0.328
High activity	6 (0.6)	6 (0.6)	16 (1.0)	16 (0.9)	12 (0.8)	14 (0.5)	0.348
1994–1995							
Inactive	59 (8.3)	53 (6.8)	74 (5.8)	89 (6.1)	62 (5.1)	186 (8.8)	<0.001
Low activity	288 (40.8)	326 (42.0)	555 (44.5)	632 (43.5)	548 (45.8)	1040 (49.7)	<0.001
Moderate activity	317 (45.7)	349 (46.1)	550 (45.2)	652 (45.9)	543 (46.4)	801 (39.0)	<0.001
High activity	37 (5.4)	40 (5.6)	56 (4.9)	67 (5.0)	34 (3.1)	53 (2.7)	<0.001
Current smoking, n (%)							
1986–1987	358 (47.4)	361 (44.1)	563 (42.4)	595 (38.4)	520 (40.6)	1008 (44.8)	<0.001
1994–1995	382 (55.5)	362 (48.8)	555 (46.2)	544 (39.1)	438 (38.0)	724 (35.5)	<0.001
History of myocardial infarction, n (%)^a^							
New cases between surveys	6 (0.4)	2 (0.1)	4 (0.2)	7 (0.2)	2 (0.1)	8 (0.2)	<0.001
History of angina pectoris, n (%)^a^							
New cases between surveys	6 (0.3)	7 (0.3)	9 (0.2)	11 (0.2)	11 (0.4)	19 (0.4)	0.865
History of stroke, n (%)^a^							
New cases between surveys	5 (0.5)	1 (0.2)	4 (0.3)	6 (0.3)	6 (0.4)	8 (0.4)	<0.001
Diabetes mellitus, n (%)^a^							
New cases between surveys	8 (1.1)	1 (0.2)	6 (0.5)	3 (0.2)	5 (0.4)	13 (0.6)	<0.001
Antihypertensive medications, n (%)							
1986–1987	24 (3.3)	8 (0.9)	22 (1.6)	27 (1.8)	25 (2.1)	41 (2.1)	0.038
1994–1995	32 (1.7)	20 (0.9)	38 (1.1)	53 (1.4)	37 (1.2)	82 (1.6)	0.086

Values are mean (SD) or number (percentage); the means (except age means) and percentages are adjusted for age between the surveys and 10‐year BMI change groups using linear mixed models or generalized estimating equations, respectively. AF indicates atrial fibrillation; BMI, body mass index; HDL, high‐density lipoprotein.

a Percentages are adjusted for age in 1986 to 1987 using a logistic regression model. Because of missing observations, numbers for the variables may be marginally less.

### Incidence of AF During Follow‐Up

Over a mean follow‐up of 15.7±5.5 years, 811 women (6.2%) and 918 men (7.9%) from the fourth Tromsø Study survey developed AF (Table 4). For individuals who attended the third and fourth Tromsø Study surveys and whose change in BMI over 10 years was calculated, 3.5% of women (n=260) and 8.0% of men (n=575) developed AF during follow‐up (Table 4).

**Table 4:** Incidence of AF During Follow‐Up in Those With a Single BMI Measurement (Fourth Tromsø Study Survey) and in Those for Whom Change in BMI Over 10 Years Was Calculated: The Tromsø Study

Sex	Single BMI Measurement		Individual 10‐y Change in BMI	
	No AF	AF^a^	No AF	AF^a^
Men	10 755	918 (7.9)	6583	575 (8.0)
Women	12 315	811 (6.2)	7234	260 (3.5)

AF indicates atrial fibrillation; BMI, body mass index.

a Data are given as number (percentage).

### Impact of Single‐Measurement BMI on the Risk of Future AF

**Figure 2:**
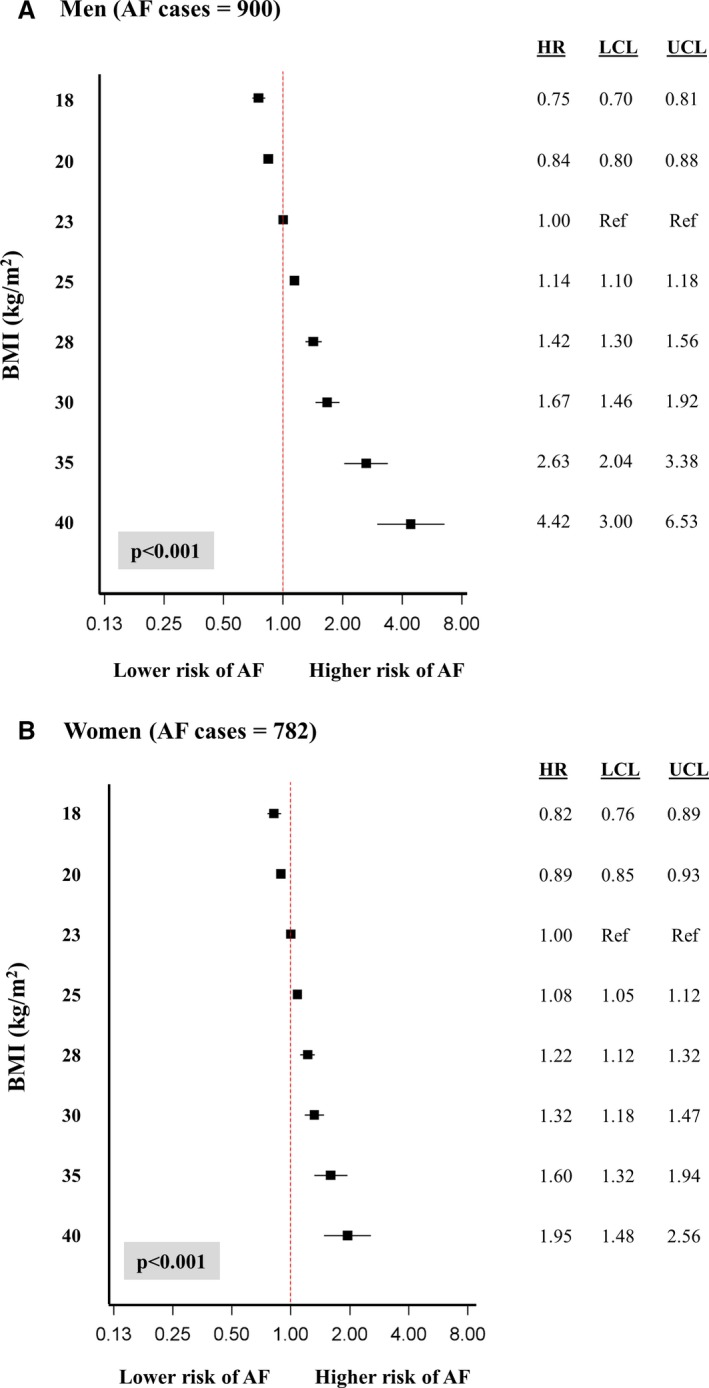
Influence of single‐measurement body mass index (BMI) on the risk of future atrial fibrillation (AF) in men (A) and women (B) in the Tromsø Study. HR indicates hazard ratio; LCL, lower confidence level; UCL, upper confidence level.

Overall, in men, lower BMI was associated with a decreased risk of future AF and higher BMI was associated with an increased risk (Figure 2A). In comparison to men with a BMI of 23.0 kg/m^2^ (reference), men with a BMI of 18.0 and 20.0 kg/m^2^ were at significantly lower risk of developing AF in the future (HR, 0.75 [95% CI, 0.70–0.81]; and HR, 0.84 [95% CI, 0.80–0.88], respectively). Men who just fell into the overweight category with a BMI of 25.0 kg/m^2^ had a 14% higher risk of incident AF than those with a BMI of 23.0 kg/m^2^ at any time during follow‐up. This risk increased significantly as BMI increased, and men who were classified as class III obese (BMI, 40.0 kg/m^2^) were at almost a 4.5‐fold higher risk of incident AF than those at the reference BMI (23.0 kg/m^2^) during follow‐up. When results for men were stratified for age (<65 years [5.1% of those who developed AF] versus ≥65 years [30.6% of those who developed AF]), the overall patterns of association with single‐measurement BMI and future AF risk were similar; lower BMI was associated with a decreased AF risk and increased BMI was associated with an increased AF risk (data not shown). However, associations were stronger for men aged <65 years compared with men aged ≥65 years, with smaller HRs at lower BMIs and larger HRs at higher BMIs. Men <65 years with a BMI of 20 kg/m^2^ had a 26% reduced risk of future AF (HR, 0.74; 95% CI, 0.68–0.80), whereas men ≥65 years with a BMI of 20 kg/m^2^ only had an 11% reduced risk (HR, 0.89; 95% CI, 0.80–0.98). At a BMI of 40 kg/m^2^, men <65 years were at >5.5 times the risk of future AF (HR, 5.59; 95% CI, 3.51–8.90), whereas men aged ≥65 years with the same BMI were at 2‐fold risk of future AF (HR, 1.97; 95% CI, 1.12–3.44).

Overall for women, a significant relationship was found between BMI and future AF (Figure 2B). A similar pattern was demonstrated in women as for men, although associations for higher BMIs were not as strong. BMIs of 18.0 and 20.0 kg/m^2^ were associated with an 18% and 11% decreased risk of future incident AF, respectively, compared with women at the reference BMI (23.0 kg/m^2^). BMIs of ≥25.0 kg/m^2^ were associated with a significantly increased risk of incident AF at any time during follow‐up; those who were classified as class III obese (BMI, 40.0 kg/m^2^) were at almost double the risk (HR, 1.95; 95% CI, 1.48–2.56).

#### Sensitivity analyses

##### Potential mediating factors

Results of sensitivity analyses testing different models of association in men and women separately with and without potential mediating factors of the relationship between BMI and incident AF are presented in Table S1. Despite removal of potential mediating factors, such as physical activity levels, current smoking, cholesterol levels, prior CVD, concurrent diabetes mellitus, and antihypertensive medication use, patterns of association were not markedly different between the models and remained highly significant (all *P*<0.001).

##### AF type

Findings of sensitivity analyses stratifying results by AF type (paroxysmal/persistent versus permanent) are presented in Table 5. For those with paroxysmal/persistent AF, no significant association between single‐measurement BMI and future AF was demonstrated (potentially because of lack of study power). For individuals with permanent AF, clear and significant associations between reduced BMI and reduced risk of future AF and between increased BMI and increased risk of future AF were demonstrated. Patterns of association were stronger in men than in women; a BMI of 40 kg/m^2^ was significantly associated with a 9‐fold increased risk in men and a 3‐fold increased risk in women of future AF development.

**Table 5:** Sensitivity Analyses Stratifying the Association of Single‐Measurement BMI With the Risk of Future Development of AF by AF Type

BMI, kg/m^2^	Paroxysmal/ Persistent AF		Permanent AF	
	HR (95% CI)		HR (95% CI)	
	Men	Women	Men	Women
18	0.90 (0.77–1.06)	0.91 (0.81–1.03)	0.52 (0.45–0.61)	0.72 (0.64–0.81)
20	0.94 (0.86–1.03)	0.94 (0.88–1.02)	0.68 (0.62–0.74)	0.82 (0.77–0.88)
23	1.00 (Reference)	1.00 (Reference)	1.00 (Reference)	1.00 (Reference)
25	1.04 (0.98–1.11)	1.04 (0.99–1.09)	1.30 (1.22–1.37)	1.14 (1.09–1.19)
28	1.11 (0.95–1.29)	1.10 (0.98–1.24)	1.91 (1.65–2.22)	1.39 (1.24–1.56)
30	1.15 (0.93–1.44)	1.14 (0.97–1.35)	2.47 (2.01–3.05)	1.59 (1.35–1.86)
35	1.28 (0.88–1.89)	1.25 (0.94–1.67)	4.73 (3.31–6.75)	2.20 (1.68–2.90)
40	1.41 (0.83–2.40)	1.38 (0.92–2.07)	9.03 (5.45–14.95)	3.06 (2.08–4.51)
AF cases, n	452	387	407	342
*P* value	0.202	0.121	<0.001	<0.001

AF indicates atrial fibrillation; BMI, body mass index; CI, confidence interval; HR, hazard ratio.

### Impact of Individual 10‐Year Change in BMI on Risk of Future AF

**Figure 3:**
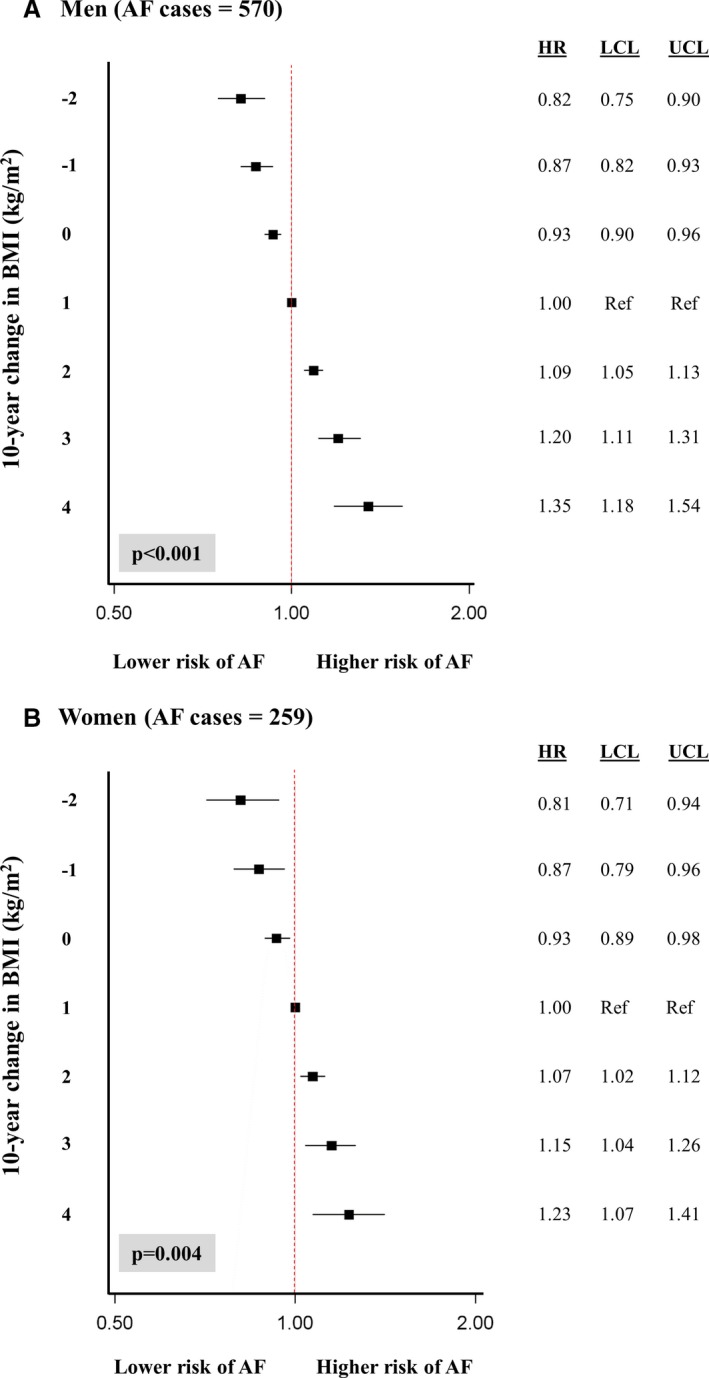
Influence of individual 10‐year change in body mass index (BMI) on the risk of future atrial fibrillation (AF) in men (A) and women (B) in the Tromsø Study. HR indicates hazard ratio; LCL, lower confidence level; UCL, upper confidence level.

Overall, in men, a decrease in BMI over 10 years was associated with a decreased risk of future AF and an increase in BMI was associated with an increased risk of AF development during follow‐up (Figure 3A). Maintenance of BMI over time was similarly associated with a significantly decreased risk of future AF of 7%. For women, the patterns of association for BMI change over time were similar to those seen for men (Figure 3B). An increase in BMI over a 10 year period of 3 or 4 kg/m^2^ was significantly associated with an increased AF risk of 15% (HR, 1.15; 95% CI, 1.04–1.26) and 23% (HR, 1.23; 95% CI, 1.07–1.41), respectively (Figure 3B).

#### Sensitivity analyses

##### AF type

Analyses of the association of change in BMI over time with risk of future AF stratified by AF type are presented in Table 6. For men with paroxysmal/persistent AF, maintenance or a reduction in BMI over 10 years significantly reduced the risk of future AF, and increased BMI significantly increased the risk of future AF. The pattern was the same in women, although significance was not reached (*P*=0.058). For those with permanent AF, maintenance or reduction in BMI over 10 years significantly reduced the risk of future AF and an increase in BMI by ≥2 units significantly increased the risk of future AF in both men and women (*P*=0.005 and *P*=0.012, respectively). HRs for men and women with permanent AF were similar.

**Table 6:** Sensitivity Analyses Stratifying the Association of 10‐Year Change in BMI With the Risk of Future Development of AF by AF Type

10‐Year Change in BMI, kg/m^2^	Paroxysmal/ Persistent AF		Permanent AF	
	HR (95% CI)		HR (95% CI)	
	Men	Women	Men	Women
−2	0.77 (0.64–0.93)	0.84 (0.70–1.01)	0.83 (0.73–0.95)	0.74 (0.59–0.94)
−1	0.84 (0.74–0.95)	0.89 (0.79–1.00)	0.88 (0.80–0.96)	0.82 (0.70–0.96)
0	0.92 (0.86–0.98)	0.94 (0.89–1.00)	0.93 (0.89–0.98)	0.91 (0.84–0.98)
1	1.00 (Reference)	1.00 (Reference)	1.00 (Reference)	1.00 (Reference)
2	1.09 (1.02–1.16)	1.06 (1.00–1.13)	1.08 (1.02–1.15)	1.10 (1.02–1.19)
3	1.19 (1.05–1.35)	1.12 (1.00–1.27)	1.19 (1.05–1.35)	1.22 (1.04–1.42)
4	1.30 (1.08–1.56)	1.19 (0.99–1.42)	1.32 (1.09–1.61)	1.34 (1.07–1.70)
5	1.41 (1.10–1.81)	1.26 (0.99–1.60)	1.49 (1.13–1.97)	1.48 (1.09–2.02)
AF cases, n	290	159	268	96
*P* value	0.006	0.058	0.005	0.012

AF indicates atrial fibrillation; BMI, body mass index; CI, confidence interval; HR, hazard ratio.

## Discussion

In the present study, we expand the current body of knowledge on the impact of BMI on risk of AF by analyzing BMI change over time. Within a large population cohort, we tested and confirmed sex differences in the influence of BMI on future risk of AF. In both men and women, higher BMI (beyond that classified as “normal”) was significantly and independently associated with an increased risk of future AF, and having a lower BMI conferred a decreased risk, during an average follow‐up of almost 16 years. These associations were stronger in men; a BMI of 40.0 kg/m^2^ increased the risk of future AF incidence by ≈4.5‐fold in men compared with almost 2‐fold in women. In addition, associations were stronger in men aged <65 years than in those aged ≥65 years. In both men and women, regardless of baseline measurement, those who decreased or maintained their BMI over a 10‐year period decreased their risk of future AF. In addition, those who increased their BMI by ≥2 kg/m^2^ over a 10‐year period increased their risk of future AF. All results were independent of age, baseline BMI (for change analyses only), blood pressure, cholesterol levels, current smoking, physical activity levels, history of CVD, concurrent diabetes mellitus, and current antihypertensive medication use. Removal of potential mediators of the relationship between BMI and incident AF did not substantially change the models and did not affect significance. Stratification of results by AF type revealed that single‐measurement BMI was not significantly associated with future AF risk in men and women with paroxysmal/persistent AF. Lower BMI was associated with reduced risk of future AF and higher BMI was associated with increased risk of future AF in both men and women with permanent AF, although associations were stronger in men than in women. Change in BMI over time in those with paroxysmal/persistent AF was significantly associated with future AF risk in men only, not women. For those with permanent AF, change in BMI over time was significantly associated with future AF risk in both men and women, with decreasing BMI associated with decreased AF risk and increasing BMI associated with an increased future AF risk.

Overweight and obesity are well‐established major CVD risk factors,^29^ and previous population studies have demonstrated that overweight and obesity are significantly and independently associated with increased AF risk.^9^, ^12^, ^13^, ^17^, ^18^, ^30^, ^31^, ^32^, ^33^, ^34^, ^35^ Few studies have, however, assessed the influence of BMI across the spectrum from underweight to severely obese, and most focus only on obesity. Furthermore, none have conducted statistical analyses using fractional polynomials, which allows the comprehensive identification of patterns of association. Results of a meta‐analysis of data from 9 cohort studies and 14 case‐control studies suggested that there was a 19% to 29% excess risk of AF associated with every 5 kg/m^2^ increase in BMI in the general population (odds ratio, 1.29 [95% CI, 1.23–1.36]; and odds ratio, 1.19 [95% CI, 1.13–1.26], respectively).^36^ Only 3 of the included studies, however, provided sex‐specific estimates.^36^ Recent results from a substudy of the Biomarcare Consortium involving data from 4 community‐based European studies (FINRISK [Finnish National Risk Factor Survey], DanMONICA [Danish Monitoring of Trends and Determinants in Cardiovascular Disease], Moli‐sani, and Northern Sweden), including data from 79 793 individuals, found that among the classic risk factors, BMI explained the largest proportion of AF risk. Sex differences were seen for risk associations of BMI (HR per SD increase was 1.18 [95% CI, 1.12–1.23] in women versus 1.31 [95% CI, 1.25–1.38] in men; interaction *P*=0.001).^37^ Therefore, the current study adds to the body of knowledge by comprehensively determining sex differences in the impact of BMI on future incident AF (including providing BMI‐specific HRs) and confirms that associations between BMI and future development of AF are significant in both sexes but differ in size.

We analyzed data from a large sex‐balanced population sample with extensive and relatively recent mean follow‐up of almost 16 years. We also investigated the impact of BMI across the spectrum from underweight to severely obese and the impact of change in BMI over time. Our results suggest that BMI has a sex‐specific influence on the risk of future incident AF; higher BMI potentially has a greater influence in men than in women. These differences may be because of differences in the cause and pathophysiological features of AF that exist between the sexes.^21^, ^38^ BMI asserts its effect on the development of AF, partly through the development of fibrosis.^39^ Fibrosis resulting from higher BMI has previously been demonstrated to be more severe in men than in women.^39^ Our finding that decreasing BMI over time results in a significantly decreased risk of future AF development supports the findings of others that structural and electrical cardiac remodeling is reversible and reversibility can be induced with aggressive risk factor modification, including the reduction of BMI in overweight or obese individuals.^40^

The findings of this study carry important clinical implications that differ for men and women. Although substantial effort has been invested in identifying the associations of overweight and obesity on AF development, little work has been conducted on the beneficial effect of weight reduction in the primary prevention of AF. Berkovitch and colleagues did, however, demonstrate that in a cohort of middle‐aged adults (mean age, 49±11 years; 73% men) attending annual screening at a tertiary medical center, during 6±4 years of follow‐up, each unit reduction in BMI was associated with a significant 7% reduction in the risk for the occurrence of a first AF event (HR, 0.93; 95% CI, 0.88–0.99; *P*=0.019).^34^ Further understanding of sex‐specific differences in the influence of BMI on incident AF will assist in the primary prevention of this disease and has the potential to contribute to more specific and personalized (sex‐specific) preventive strategies. Interventions to promote normal weight should be encouraged for those at high risk, with even modest reductions in population BMI likely to have a significant effect on the public health burden of AF.

### Limitations

There exist some limitations of this study that require comment. First, baseline data were collected via self‐report, which may have underestimated or overestimated the prevalence of smoking, level of physical activity, comorbidities, and antihypertensive medication use within this cohort. Second, detailed data on prescribed drug therapy and comorbidities, which may or may not have influenced the future development of AF or may have been more common in one sex over the other (potentially having a protective or promoting effect on AF development), were also not collected. Many changes in prescribed medical therapies and comorbidities may have occurred during follow‐up that also could have influenced the risk of incident AF. Third, we cannot be sure of changes in BMI that took place within individuals during follow‐up that may have influenced (negatively or positively) the risk of future AF. Fourth, we also cannot be sure whether the biomedical risk factors and comorbidities considered in the analysis of single‐measurement BMI are potential mediators or confounders to the relationship between BMI and future AF, although it is likely that they are a combination of both and removal revealed little change to the models of association identified. Although BMI is a conventional measurement, it does not take into account muscle mass and, therefore, some participants may not actually have excess fat, although these individuals would be expected to have lower incidence of AF. Furthermore, no data on body fat percentage or pericardial fat were collected in the current study. We were also not able to analyze the influence associations of BMI in symptomatic versus asymptomatic AF because these data on symptoms were not collected. Exclusion of participants with missing data points reduced the sample size and may have resulted in the loss of some cases of AF, although not many participants with missing data existed. There may also have been individuals who developed AF but were not diagnosed during follow‐up (eg, those with silent asymptomatic AF). Finally, associations were not adjusted for cardiorespiratory fitness, which may have influenced the risk of future AF development.

## Conclusions

Within a large population cohort, higher BMI was significantly and independently associated with an increased risk of future AF that was larger for men than women. Changes in BMI over 10 years influenced the risk of AF in both men and women. Weight maintenance/reduction strategies should potentially be different for women and men but should form part of a lifetime approach to the primary prevention of AF for both sexes. Future research is needed to confirm our findings and to understand the responsible mechanisms.

## Sources of Funding

This work was supported by the Norwegian Health Association in Troms County. Ball (APP1112829) is supported by the National Health and Medical Research Council of Australia and by a Postdoctoral Fellowship (Award Reference 100950) from the National Heart Foundation of Australia. This work was supported in part by the Victorian Government's Operational Infrastructure Support Program.

## Disclosures

None.

## Supporting information


**Table S1.** Models Testing the Influence of Single Measurement BMI on the Risk of Future AF in Men and Women in the Tromsø Study With Progressive Inclusion of Potential Mediators to the Relationship
