# Supplementary material for: Sex Differences in the Impact of Body Mass Index on the Risk of Future Atrial Fibrillation: Insights From the Longitudinal Population‐Based Tromsø Study
Source: J Am Heart Assoc. 2018 Apr 19;7(9):e008414. doi: 10.1161/JAHA.117.008414 (PMC6015294; doi:10.1161/JAHA.117.008414)
Supplement: Supplementary file 1 — Table S1. Models Testing the Influence of Single Measurement BMI on the Risk of Future AF in Men and Women in the Tromsø Study With Progressive Inclusion of Potential Mediators to the Relationship [file JAH3-7-e008414-s001.pdf]

SUPPLEMENTAL MATERIAL

Table S1. Models testing the influence of single measurement BMI on the risk of future AF in men and women in the Tromsø Study with progressive inclusion of potential mediators to the relationship.

	**Model 1***	**Model 2***	**Model 3 (presented)***
**MEN**			
**BMI (kg/m^2)**	**HR (95% CI)**	**HR (95% CI)**	**HR (95% CI)**
18	0.72 (0.68-0.77)	0.73 (0.68-0.78)	0.75 (0.70-0.81)
20	0.81 (0.78-0.85)	0.82 (0.79-0.86)	0.84 (0.80-0.88)
23	*1.00 (reference)*	*1.00 (reference)*	*1.00 (reference)*
25	1.16 (1.13-1.20)	1.16 (1.12-1.19)	1.14 (1.10-1.18)
28	1.50 (1.38-1.63)	1.47 (1.36-1.60)	1.42 (1.30-1.56)
30	1.80 (1.60-2.03)	1.76 (1.56-1.98)	1.67 (1.46-1.92)
35	3.02 (2.42-3.76)	2.88 (2.29-3.61)	2.63 (2.04-3.38)

40	5.47 (3.89-7.69)	5.09 (3.58-7.22)	4.42 (3.00-6.53)
**AF cases (n)**	918	911	900
**p value**	<0.001	<0.001	<0.001
**WOMEN**			
**BMI (kg/m^2)**	**HR (95% CI)**	**HR (95% CI)**	**HR (95% CI)**
18	0.77 (0.72-0.83)	0.76 (0.71-0.82)	0.82 (0.76-0.89)
20	0.86 (0.82-0.89)	0.85 (0.81-0.89)	0.89 (0.85-0.93)
23	*1.00 (reference)*	*1.00 (reference)*	*1.00 (reference)*
25	1.11 (1.08-1.14)	1.12 (1.08-1.15)	1.08 (1.05-1.12)
28	1.30 (1.21-1.39)	1.31 (1.22-1.41)	1.22 (1.12-1.32)
30	1.44 (1.30-1.59)	1.47 (1.32-1.63)	1.32 (1.18-1.47)
35	1.87 (1.57-2.22)	1.93 (1.61-2.30)	1.60 (1.32-1.94)

40	2.42 (1.90-3.09)	2.53 (1.97-3.25)	1.95 (1.48-2.56)
**AF cases (n)**	811	799	782
**p value**	<0.001	<0.001	<0.001

BMI = body mass index; HR = hazard ratio; CI = confidence interval; AF = atrial fibrillation

***Model 1:** Cox regression with AF as the dependent variable, fractional polynomials of BMI as the main predictor and fractional polynomials of age as a covariate; **Model 2:** Model 1 with additional adjustment for current smoking and physical activity; **Model 3 (presented model):** Model 2 with additional adjustment for heart attack, angina, stroke, diabetes, systolic blood pressure, total cholesterol, HDL cholesterol, triglycerides and antihypertensive medication.
